# Impact of root canal preparation using two single-file systems on the intra-radicular microbiome of teeth with primary apical periodontitis

**DOI:** 10.1007/s00784-024-05544-2

**Published:** 2024-02-08

**Authors:** Rodrigo Rodrigues Amaral, Robert M. Love, Tiago Braga, Maria I. Souza Côrtes, Caio T. C. C. Rachid, Isabela N. Rôças, José F. Siqueira

**Affiliations:** 1 College of Medicine and Dentistry, James Cook University, 1/14-88 McGregor Rd, Building D1, 2nd Floor, Campus Smithfield, Smithfield, Cairns, QLD 4878 Australia; 2 School of Medicine and Dentistry, Griffith University, Gold Coast, Queensland Australia; 3 Department of Dentistry, Pontifícia Universidade Católica de Minas Gerais, Belo Horizonte, Minas Gerais Brazil; 4 Laboratory of Biotechnology and Microbial Ecology, Institute of Microbiology Prof. Paulo de Góes, Federal University of Rio de Janeiro, Rio de Janeiro, RJ Brazil; 5 Department of Dental Research, Faculty of Dentistry, Iguaçu University (UNIG), Nova Iguaçu, RJ Brazil

**Keywords:** 16S ribosomal RNA sequencing, Apical periodontitis, Endodontic infection, High-throughput sequencing, Root canal preparation

## Abstract

**Objectives:**

This study aimed to describe the effects of two single-file systems on the diversity of the endodontic microbiome of teeth with primary asymptomatic apical periodontitis.

**Materials and methods:**

The root canals from single-rooted teeth with apical periodontitis were prepared using either the Reciproc Blue (RB) or the XP-endo Shaper (XPS) instrument system. The latter was followed by a supplementary step with the XP-endo Finisher (XPF) instrument. For irrigation, 5.25% sodium hypochlorite was used. Root canal samples were taken at the baseline (S1), after preparation (S2), and after the supplementary step (S3). DNA was extracted and subjected to high-throughput sequencing using the MiSeq Illumina platform.

**Results:**

Samples from 10 teeth from the RB and 7 from the XPS group were subjected to DNA sequencing. Initial samples differed significantly from post-preparation samples in bacterial diversity, with no significant difference when comparing the two instrument systems. The most dominant phyla in S2 were Bacteroidetes, Proteobacteria, Firmicutes, Fusobacteria, and Actinobacteria. The same phyla were found to dominate baseline samples and samples taken after using XPF, but with differences in the ranking of the most dominant ones. At the genus level, the most dominant genera identified after RB instrumentation were *Bacteroidaceae* [G-1], *Fusobacterium*, and *Staphylococcus*, while the most dominant genera after XPS instrumentation were *Fusobacterium* and *Porphyromonas.* These genera were also dominant in the initial samples.

**Conclusions:**

Both treatment protocols had measurable effects on the root canal microbial diversity, with no significant differences between them. Most of the dominant taxa involved in the primary infection and probably in the aetiology of apical periodontitis were eliminated or substantially reduced.

**Clinical relevance:**

The most dominant taxa that persisted after instrumentation were *Fusobacterium, Porphyromonas*, *Staphylococcus*, and *Bacteroidaceae* [G-1].

## Introduction

The main goal of disinfection of the infected root canal system is to reduce the bacterial counts to a level compatible with the healing of periradicular tissues, which is paramount for a positive endodontic treatment outcome [1]. Root canal instruments with different concepts and designs have been introduced to remove the highly infected inner layer of dentin from the root canal walls [2] and promote the disruption of bacterial biofilms while maintaining the original canal shape [3–5]. Overcoming the complex anatomy of a root canal and rendering the canal bacteria-free is a challenging task irrespective of the introduction of new instruments, techniques, and irrigants [6–8].

Over the last decade, single-file nickel-titanium (NiTi) instrumentation systems have become widely used by clinicians for cleaning and shaping root canals. Root canal preparation with a single instrument may be time-saving and cost-effective compared to multiple rotary instrument systems. Reciproc Blue (RB; VDW, Munich, Germany) is a single-file system based on reciprocating motion. According to the manufacturer, alteration of the molecular alloy structure by blue heat treatment enhances the flexibility and fatigue resistance of the instrument and in combination with a non-cutting tip improves the centring ability and reduces canal transportation. The cutting performance is attributed to the S-shaped cross-section, taper, and cutting angles [9, 10].

XP-endo Shaper (XPS; FKG Dentaire, La Chaux-de-Fonds, Switzerland) is a single-file system manufactured with MaxWire NiTi alloy (Martensite-Austenite electropolish-fleX). It is operated in continuous rotation and when exposed to the body temperature, it expands from an initial taper of 0.01 mm/mm in the “M” phase to a taper of 0.04 mm/mm and is expected to reach more areas of the canal than conventional instruments. Its booster tip has six cutting edges that allow the instrument to enlarge canals from size #15 to #30, while still maintaining the original canal pathway [11, 12].

The XP-endo Finisher file (XPF) (FKG Dentaire, La Chaux-de-Fonds) is also made from MaxWire NiTi alloy and consists of a small core non-tapered instrument, with tip size #25. The instrument is straight at room temperature, but assumes a spoon-like shape at body temperature, which makes it to expand its reach to touch the canal walls and agitate the irrigant solution. The XPF instrument was introduced as a supplementary step after root canal instrumentation to improve cleaning and disrupt residual bacterial biofilms while still preserving dentin [5].

Over the past two decades, the study of the microbiome in infected root canals has moved to sophisticated molecular biology techniques [13, 14]. High-throughput sequencing (HTS) (also known as next-generation sequencing) technologies belong to the fifth generation of endodontic microbiological studies [14] and have been widely used to evaluate the microbiome associated with different manifestations of apical periodontitis [14–17]. These methods provide open-ended analyses of endodontic infections with a deep sequencing depth that has revealed a bacterial diversity much higher than previous methodologies.

Identification of the bacterial taxa by methods used in the first four generations of endodontic microbiology studies revealed that Gram-positive bacteria are the most prevalent and dominant taxa in samples taken immediately after treatment procedures [18–21]. Not many studies have used HTS methods to this purpose [22–24], but these studies have shown that not only Gram-positive, but also Gram-negative and uncultivated bacteria may be detected in the root canals after preparation. Because residual bacteria detected in the canals at the time of filling represent an important risk factor for posttreatment apical periodontitis [25–27], it is very important to determine the identity of such persistent taxa using highly sensitive technology.

The present study evaluated the in vivo effects of two different single-file systems and a supplementary approach on the root canal microbiome of asymptomatic teeth with apical periodontitis. The MiSeq Illumina HTS platform was employed to describe the microbiome and compare the bacterial diversity before and after instrumentation.

## Materials and methods

### Patients and case selection

The study protocol was approved by the Institutional Review Board of the Pontifical Catholic University of Minas Gerais, Brazil (Ethics Committee approval CAAE 9713.1918.7.0000.5137) and performed in accordance with the principles stated in the Declaration of Helsinki.

### Study design

**Fig. 1:**
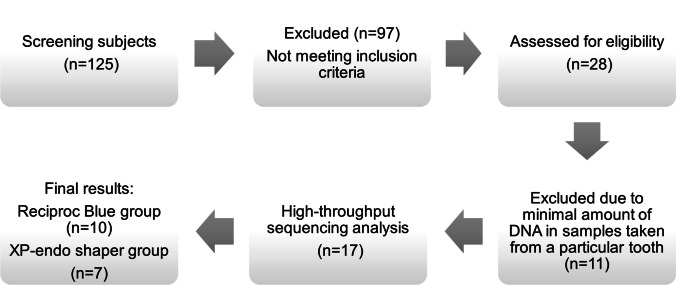
Flowchart of the study design from patient screening to assessment

Figure 1 depicts the recruitment flowchart from patient selection to HTS assessment. One hundred and twenty-five patients who had signed a free and informed consent form from a previous study [28] were selected. The inclusion criteria were patients with no systemic disease, clinical and radiographic evidence of asymptomatic primary apical periodontitis of a mature permanent single root and single canal tooth, negative pulp sensibility test, and presence of a carious lesion and intact pulp chamber walls. Exclusion criteria were teeth with extensive caries that did not permit rubber dam placement, teeth with previous endodontic treatment, presence of root or crown fracture, periodontal pocket deeper than 4 mm and patients who received antibiotic therapy in the last 3 months.

Teeth from 28 patients met the inclusion and exclusion criteria. Radiographs of the teeth were recorded from the orthogonal, mesial, and distal positions (EZSensor, Vatech, Busan, Republic of Korea) with 20° horizontal angle variation for mesial and distal positions using an intra-oral digital EZSensor holder (Vatech). Measurements were performed using the digital software EZDent-I (Vatech). Root canals presenting a buccolingual diameter nearly four times greater than the mesiodistal diameter in this study were classified as long oval or flat.

### Sample taking procedures

Samples were collected from the root canals under strict asepsis as described previously [28]. The operative field was disinfected by a two-step disinfection protocol with the sequential use of 3% hydrogen peroxide (Lenzafarm, Belo Horizonte, MG, Brazil) and 5.25% sodium hypochlorite (NaOCl) (Lenzafarm) both before and after preparation of the access cavity. The second disinfection step was performed after access cavity preparation and included the pulp chamber. Next, a 5% sodium thiosulfate solution was used to inactivate the NaOCl, and sterility control samples were taken from the internal surfaces of the cavosurface angle of the access cavity using sterile paper points which were transferred aseptically to a cryotube containing ultrapure water and immediately frozen at − 80 °C. For the continuation of the tooth in the study, control samples had to test negative, and the initial sample collected from the root canal, which served as the baseline (S1), had to test positive for the presence of bacteria in an end-point polymerase chain reaction (PCR) using universal bacterial primers [28].

To obtain root canal microbial samples, sterile 5% sodium thiosulfate solution was used to irrigate the root canal, and a K-type hand file #15 (Dentsply Maillefer, Ballaigues, Switzerland) was placed 1 mm short of the root apex as determined radiographically and confirmed by an electronic apex locator (Root ZX II; J Morita, Irvine, CA). The file was gently used in a circumferential filing motion to suspend the canal contents. Sterile paper points were consecutively placed in the canal to a level approximately 1 mm short of the radiographic root apex to soak up the fluid and left for 1 min. The paper points were then inserted in tubes containing 1 mL of sterile ultrapure water and homogenized in a vortex for 3 min. Subsequently, the paper points were removed with sterile tweezers, and the tubes were stored in a freezer at − 80 °C. The same procedures were performed to obtain samples (S2, S3) after chemomechanical preparation.

### Root canal treatment procedures

Root canals were prepared either using RB (*n* = 14) or XPS (*n* = 14) instruments. Preparation was completed in a single visit by a single endodontic specialist. After taking S1 samples in both groups, the root canal was irrigated with 5 mL of 5.25% NaOCl. A glide path was established using a size #15 K-file (Dentsply Maillefer, Ballaigues, Switzerland); to establish apical foramen patency, an electronic apex locator (Root ZX II; J Morita, Irvine, CA, USA) was initially used, and the working length confirmed with a periapical radiograph.

In the RB group, a file of #25 size and 0.08 taper was introduced into the canal and used as recommended by the manufacturer and powered by an electric motor (VDW Silver; VDW). The instrument was used in a slow in-and-out motion, approximately 3 mm in amplitude in the apical direction for 10 s, using a gentle brushing motion against the canal wall. After three apical movements, the file was removed from the canal and cleaned. The canal was irrigated with 5 mL of 5.25% NaOCl using 30-gauge NaviTip needles (Ultradent, South Jordan, UT) up to 2 mm short of the working length (WL). This procedure was performed twice until the WL was reached. After preparation, the apical foramen patency was checked with a size #20 K-type hand file [28]. Next, the root canal was irrigated with 5 mL 17% ethylenediaminetetraacetic acid (EDTA) and 5 mL 5.25% NaOCl to remove the smear layer. After chemomechanical preparation, the canal was flushed with 3 mL 5% sodium thiosulfate for 1 min to inactivate the 5.25% NaOCl, and a microbial sample (S2) was collected from the root canal.

For the XPS group, the instrument XPS (size #30, 0.01 taper) was used on rotation mode at 800 rpm and 1 N-cm torque as per manufacturer recommendations using an electric motor (VDW Silver; VDW). Each root canal was filled with 5 mL of 5.25% NaOCl. The file was used for 10 s with slightly up-and-down movements and gentle strokes, with an amplitude of 3 mm up to the WL. Once the file reached the WL, three cycles of 10 more up-and-down motions were applied. Irrigants were delivered using 30-gauge NaviTip needles up to 2 mm short of the WL. The same procedures employed in group 1 to remove the smear layer and to inactivate the 5.25% NaOCl were performed. Next, the S2 sample was collected from the root canal. Following this, the canals were irrigated with 5 mL of 5.25% NaOCl and the XPF instrument (size #25, taper 0.00) placed to WL and activated for 60 s on rotation mode at 800 rpm and 1 N-cm torque with a slow and gentle lengthwise up-and-down movements of 7–10 mm to contact the entire length of the canal. The root canal was then flushed with 3 mL of 5% sodium thiosulfate, and a microbial sample (S3) was collected from the root canal.

The standardized irrigation protocol produced a mean volume of 25 mL of 5.25% NaOCl per root canal, with an average exposure time of 12 min to the irrigants. Each instrument was used to prepare only one canal, and all teeth were subsequently root-filled in a single visit.

### Molecular analysis

DNA was extracted from the samples using the MiniSpin DNA extraction kit (Kasvi, São José dos Pinhais, PR, Brazil) following the manufacturer’s instructions. The 16S ribosomal RNA (rRNA) gene sequencing was performed by Neoprospecta (Florianópolis, SC, Brazil) according to the company’s protocols. The DNA sequencing was performed using primers 314F–806R, which target the hypervariable regions V3–V4, and library construction was carried out following the protocol 16S Sample Preparation Guide (Illumina).

Sequencing was carried out using the MiSeq platform with the single-end strategy. The raw sequences were processed using Mothur v.1.45.3 software (Schloss et al. 2009). The raw sequencing file was received from Neoprospecta, containing the fastq files already individualized by each sample, without adaptors and barcodes. Fastq files were converted to “.fasta” and “.qual” files using fast.info command. All fasta and all qual files were merged using merge.files command, and a “.group” file (used to map each sequence to a given sample) was created using make.group command.

Sequences were then trimmed with the command trim.seqs, using the following criteria: qwindowaverage = 25, qwindowsize = 20, minlength = 200, maxambig = 0. Sequences were then aligned against a pre-processed version of the Silva nr database v132 (passed by a virtual PCR with the primers for the hypervariable region for v3–v4 region of the 16S, used to amplify the samples). The resultant alignment was submitted to screen.seqs to remove sequences with bad alignment and to define the limits of the alignment and then to filter.seqs to remove all uninformative columns of the alignment. Then, sequences were preclustered using pre.cluster command with parameter diffs = 2. Chimeric sequences were detected using the chimera.vsearch command, with the sequences themselves as reference with the option derreplicate = t. To remove contaminants, sequences were classified using the silva.nr_v132.tax database employing an 80% confidence threshold, and those classified into Chloroplasts, Mitochondria, Archaea, Eukarya, and not assigned to any kingdom were removed. The remaining high-quality sequences were clustered into operational taxonomic units (OTUs) using dist.seqs followed by cluster command, with a 3% sequence dissimilarity cutoff, and all singletons were removed using the command split.abund. Lastly, the samples were randomly normalized to the same number of sequences (14,342). To define the bacterial taxonomic composition of the samples, sequences were classified again, using the Human Oral Microbiome Database, employing an 80% confidence threshold (HOMD_16S_rRNA_RefSeq_V15.22).

Statistical tests were run using PAST 4.0 software (Hammer et al. 2001). The OTU distribution in each sample was used for alpha-diversity calculation, as well for microbial structure analysis, which was performed using non-metric multidimensional scaling (NMDS) with Bray–Curtis distance and a two-way Permanova.

## Results

HTS analyses included 10 teeth from the RB (S1 and S2 samples) and 7 teeth from the XPS (S1 to S3 samples) groups. The other cases were excluded because there was no or only a minimal amount of DNA in any of the samples taken from a particular treated tooth. Sterility controls from all cases included in this study resulted in negative results as evaluated by end-point PCR with universal primers, and bacterial DNA was present in all initial root canal samples [28].

**Fig. 2:**
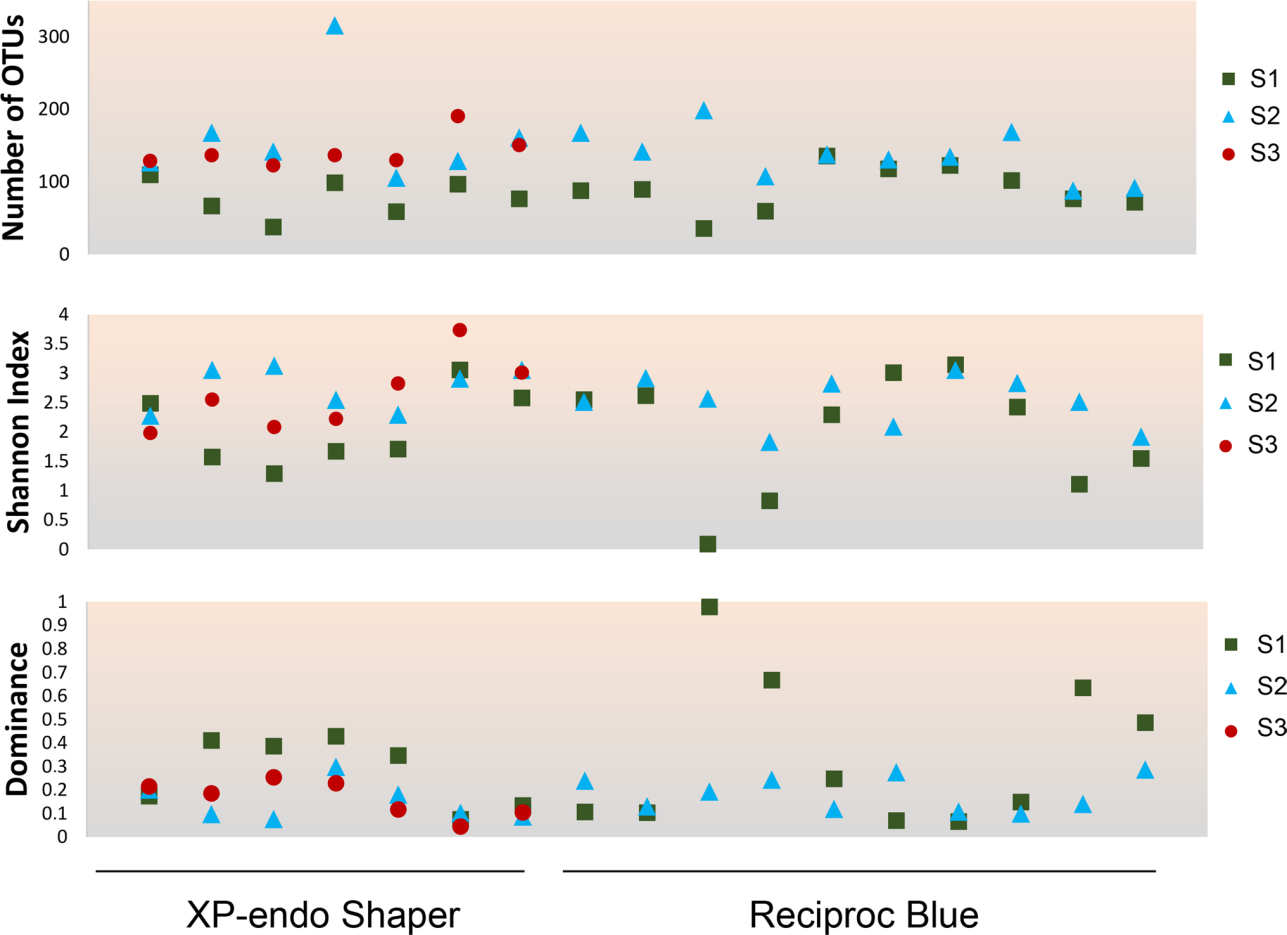
Diversity and richness calculations. Number of operational taxonomic units (OTUs), Shannon diversity index and dominance data in samples taken before and after root canal preparation with two instrument systems. Spearman correlation data for comparisons were as follows: OTU numbers vs. Dominance (*r* =  − 0.47, *p* = 0,002); OTU numbers vs. Shannon Index (*r* = 0.64, *p* = 5.73E − 06); and Shannon Index vs. Dominance (*r* =  − 0.94, *p* = 2.4128E − 19)

Figure 2 presents data from diversity and richness estimate calculations. In general, the mean number of species-level OTUs per root canal teeth in S1 was 90 (range, 36–136) in the RB group and 78 (38–110) in the XPS group. In S2, the mean number of species was 137 (range, 88–199) in the RB group and 164 (range, 106–382) in the XPS group. In the S3 samples taken after using XPF in the latter group, the mean number of species was 143 (range, 150–305). Although the number of different OTUs was increased in posttreatment samples, data for dominance was the opposite, with S1 showing higher values. Data from the Spearman correlation test showed an inverse relationship between the number of OTUs (richness) and dominance (*p* = 0.002). Thus, samples with a larger number of OTUs exhibited lower species dominance. In an infectious process, it is expected that one or a few species dominate, reducing the diversity. Indeed, the correlation between dominance and diversity was negative and very strong (*r* =  − 0.94, *p* = 2.4128E − 19).

**Fig. 3:**
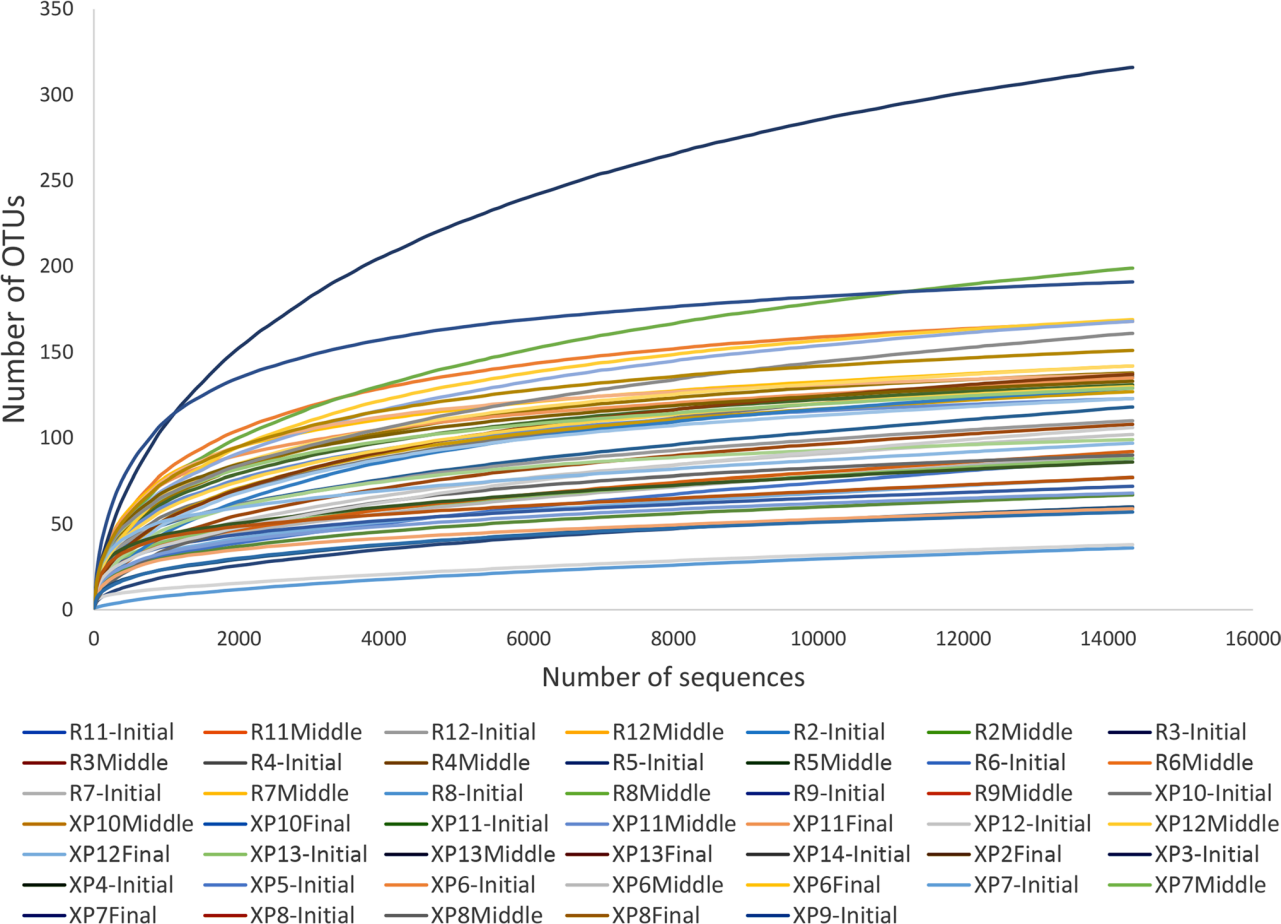
Rarefaction curves

The difference between observed and estimated OTUs indicates that there were bacterial phylotypes that remained undetected. However, the rarefaction curves disclosed a satisfactory coverage of virtually all samples (Fig. 3).

**Fig. 4:**
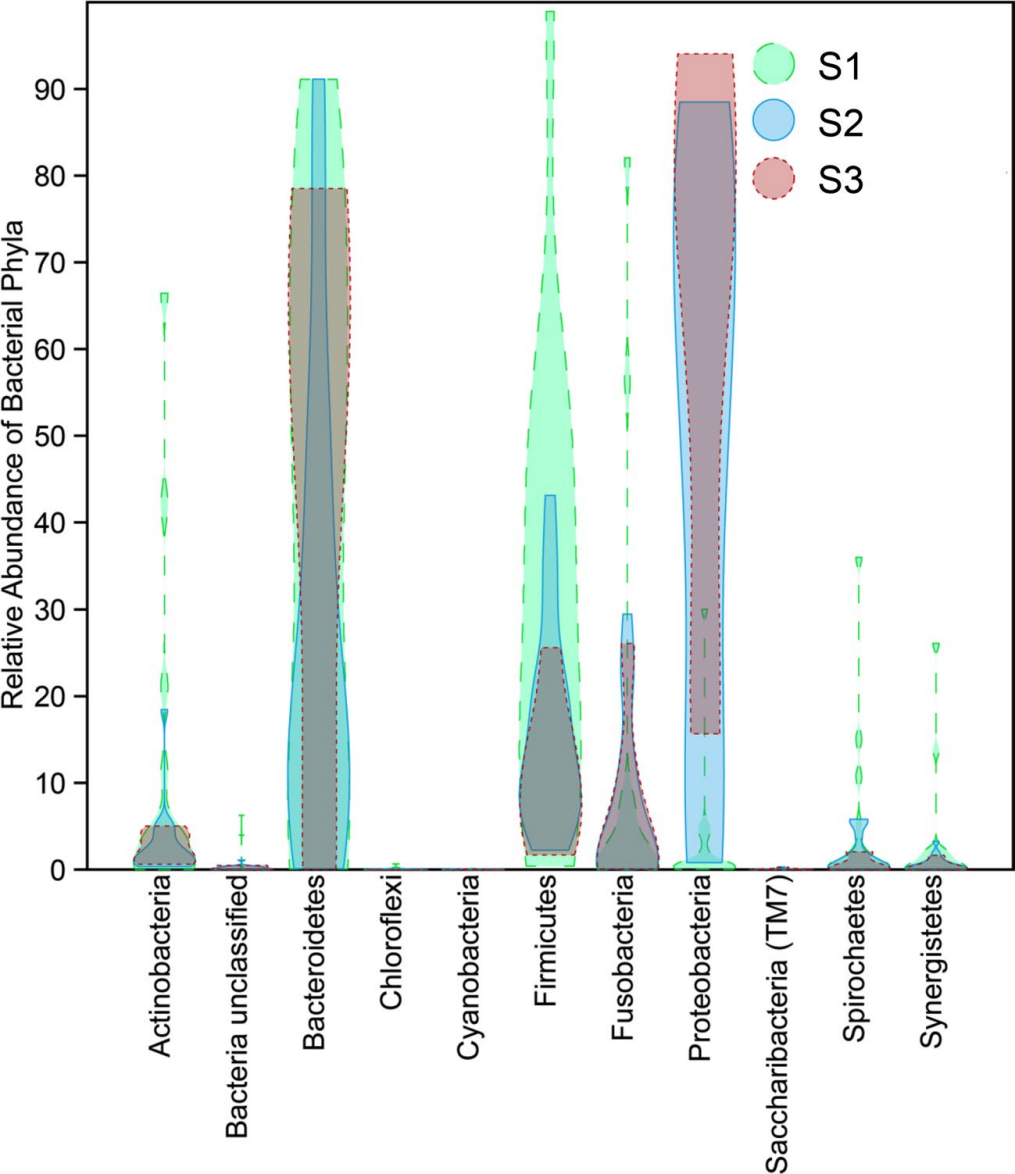
Overall relative abundance of bacterial phyla in samples taken before preparation (S1), after preparation (S2), and after the supplementary step with a finisher instrument (S3)

Overall, the most abundant phyla in S1 samples were, in decreasing order, Firmicutes, Bacteroidetes, Fusobacteria, Actinobacteria, Spirochaetes, Proteobacteria, Synergistetes, Chloroflexi, Saccharibacteria (TM7), and Cyanobacteria (Fig. 4). OTUs that could not be assigned to known phyla (unclassified) were also observed. The most dominant phyla in S2 in decreasing order were Bacteroidetes, Proteobacteria, Firmicutes, Fusobacteria, Actinobacteria, Spirochaetes, and Synergistetes. In S3, the most dominant were Proteobacteria, Bacteroidetes, Fusobacteria, Firmicutes, Actinobacteria, Spirochaetes, and Synergistetes.

**Fig. 5:**
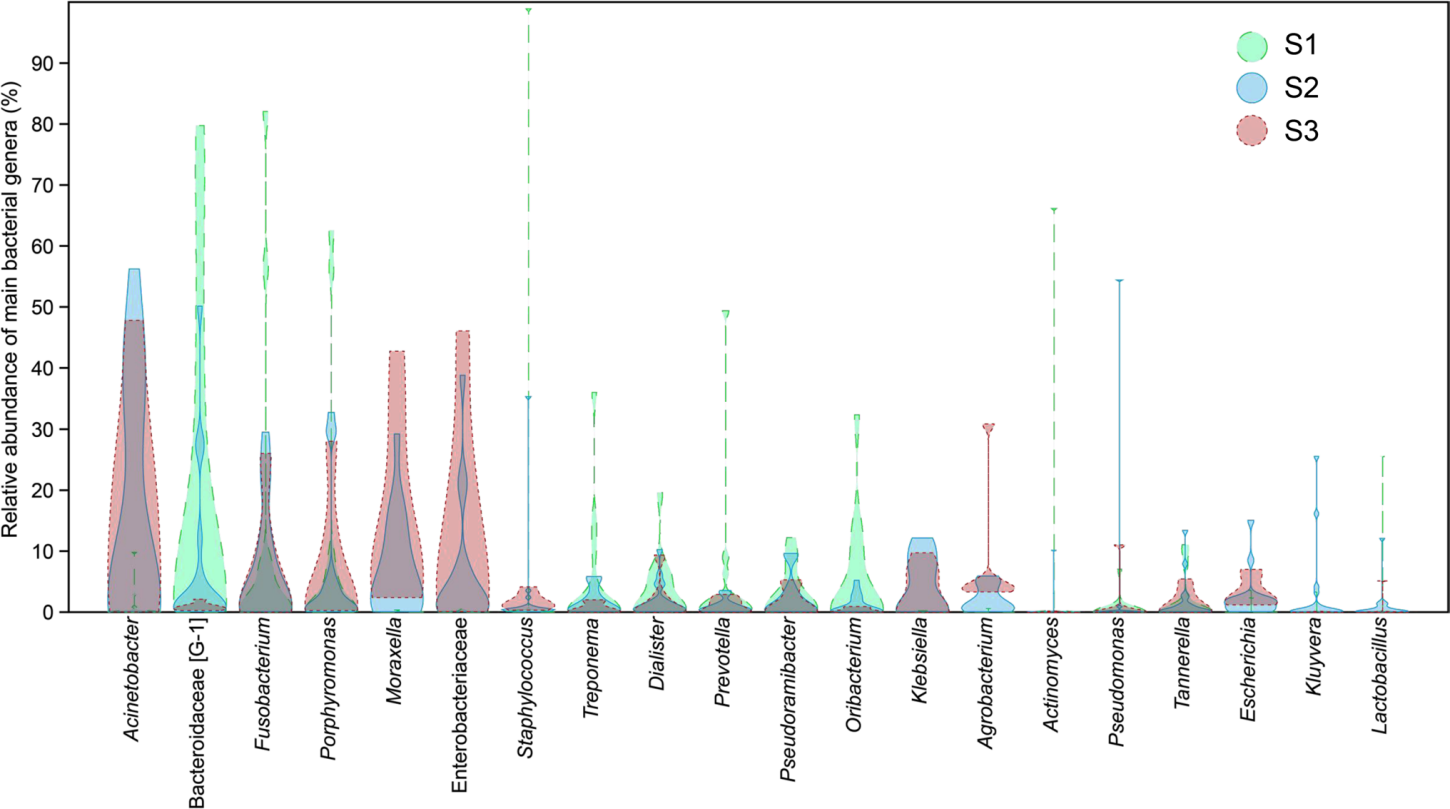
Overall relative abundance of the main bacterial genera in samples taken before preparation (S1), after preparation (S2), and after the supplementary step with a finisher instrument (S3)

**Fig. 6:**
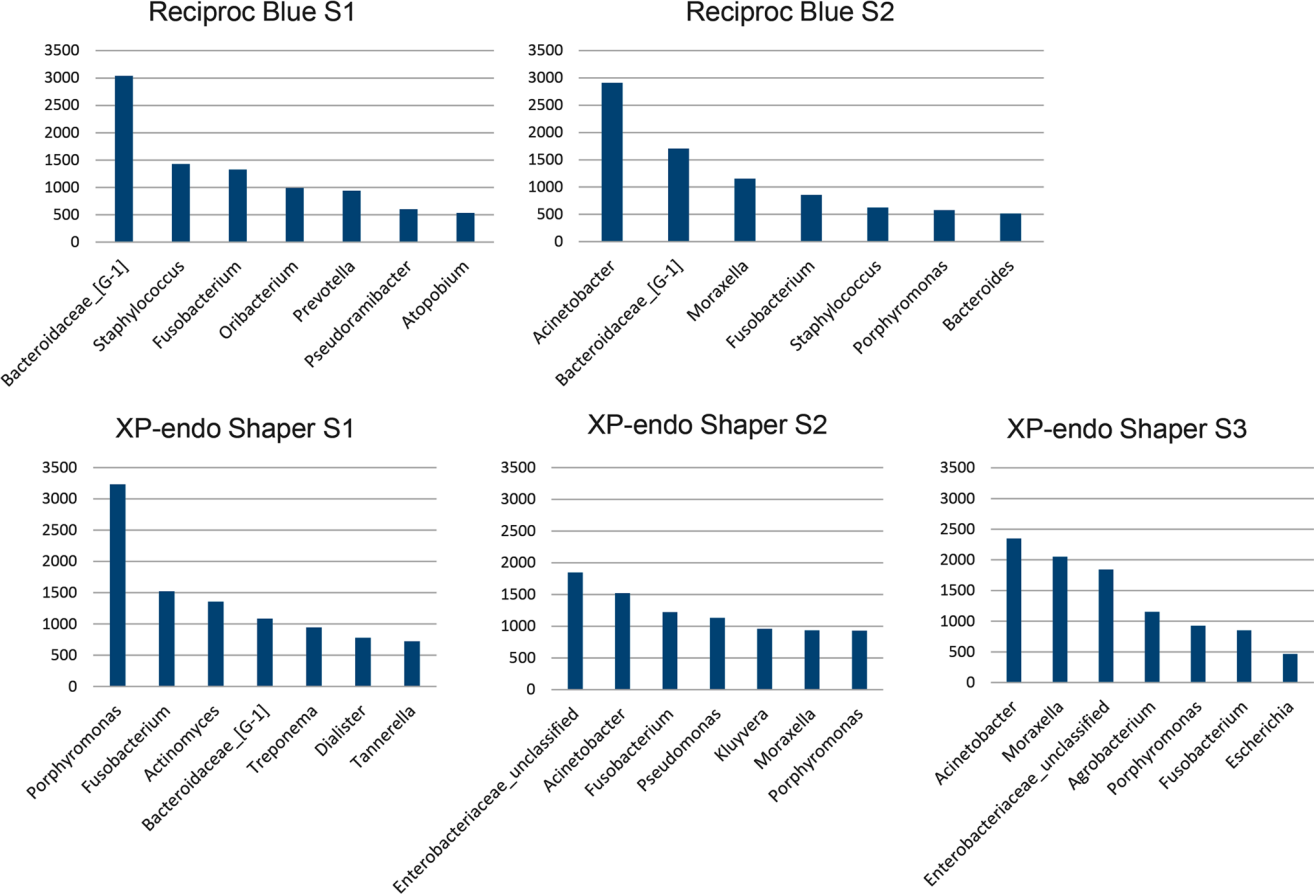
Relative abundance of the seven most dominant bacterial genera in samples taken before preparation (S1), after preparation (S2), and after the supplementary step with a finisher instrument (S3). Data according to each specific group

Figures 5 and 6 show the most dominant genera found in samples before and after preparation with the two test systems. In the RB group, the most dominant genera in S2 included *Bacteroidaceae* [G-1], *Fusobacterium*, and *Staphylococcus*, which were also amongst the most dominant in S1. *Acinetobacter* and *Moraxella* were also dominant in S2. After instrumentation with XPS, the most dominant genera detected were *Fusobacterium* and *Porphyromonas,* which were also found to dominate the microbiome in S1, as well as an Enterobacteriacea clone and *Acinetobacter*. Similar findings were observed for S3 samples taken after activation with XPF*.*

**Fig. 7:**
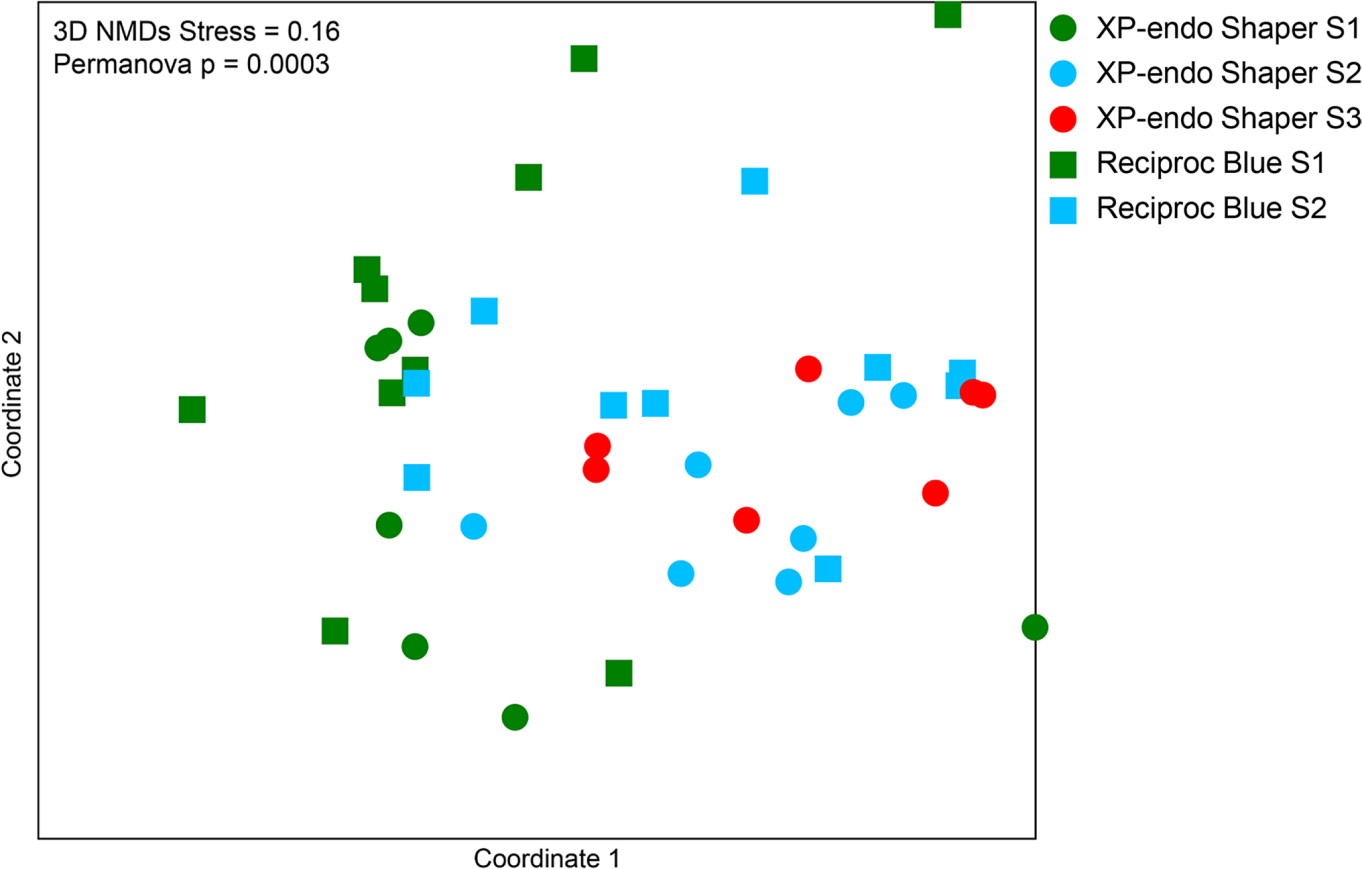
Coordinates of three-dimensional NMDS. There is a cluster of green samples (S1) on the left, while most blue (S2) and red (S3) samples are displaced to the right. This shows that the sampling time significantly affected the separation of these groups. Reciproc Blue (circles) and XP-endo Shaper (squares) groups did not differ at the community level

No significant difference in diversity occurred between S1 samples from the two groups; the same was observed for S2 comparison (two-way ANOVA and NMDS) (Fig. 7). Intragroup analysis showed a significant difference between S1 and S2 in both groups and S1 and S3 in the XPS group. Permanova followed by a pairwise test confirmed that initial samples differed significantly from post-preparation samples. However, no significant differences were observed when comparing S2 from either group with S3 from the XPS group.

## Discussion

This study used HTS technology to evaluate the microbiome associated with pre- and post-preparation root canal samples taken from infected teeth during treatment with two different instrumentation protocols. A previous study evaluating these same samples with quantitative real-time polymerase chain reaction showed a substantial reduction in total bacterial counts after instrumentation with either RB or XPS [28]. Further significant bacterial reduction was observed after using a supplementary NaOCl activation with XPF [28]. Data from the present study showed no significant differences between the two instrument systems in the bacterial diversity evaluated before and after preparation. This indicates that in addition to not being different in terms of quantitative bacterial reduction [28], instrumentation with the two systems caused a similar impact on the bacterial community structure.

Analyses of samples within groups revealed a significant difference between pre- and post-preparation communities. These findings suggest that there can be a different profile for post-preparation samples. Although the supplementary step with XPF promoted a significant additional reduction in total bacterial counts [28], it did not cause a substantial change in bacterial diversity when compared to samples taken immediately after instrumentation with XPS.

This HTS study demonstrated a high interindividual variability in the composition of the endodontic microbiome before and after preparation, with communities exhibiting high differences in species composition and abundance from subject to subject. The most dominant phyla found in the root canals after instrumentation included, in decreasing order, Bacteroidetes, Proteobacteria, Firmicutes, Fusobacteria, and Actinobacteria. These are the same phyla with most representatives and found to prevail in previous studies on the endodontic microbiome [15, 29, 30]. The very same phyla were also found to dominate baseline samples and samples taken after using XPF, but with differences in the ranking of the most dominant ones. At the genus level, the most dominant genera identified after RB instrumentation were *Bacteroidaceae* [G-1], *Fusobacterium*, and *Staphylococcus*, while the most dominant genera after XPS instrumentation were *Fusobacterium* and *Porphyromonas.* These genera were also dominant in the initial samples, indicating that, while reduced in numbers, they were not completely eliminated after instrumentation.

Whether the persistent taxa remain in numbers sufficient to compromise the long-term treatment outcome remains to be evaluated. In many cases, residual bacteria will succumb in the root canal after obturation because of the unfavourable environmental conditions induced by treatment [1]. However, because detection of residual bacteria in the root canal is a significant risk factor for posttreatment disease [25–27], it is reasonable to assume that in many cases they manage to survive and maintain periradicular inflammation. The importance of identifying the persistent taxa cannot be overstated because they can serve as targets for antimicrobial studies, such as for an intra-radicular dressing material and also as markers for predicting the treatment outcome.

Some OTUs detected in post-preparation samples were not observed in the corresponding initial samples. Indeed, in many cases, the number of distinct OTUs increased in S2 in comparison with S1. This is apparently unexpected, especially because chemomechanical procedures result in a substantial reduction in bacterial counts [31–33]. However, there are some reasonable explanations for this. Sequencing is not a quantitative approach and the fact that more OTUs were observed does not mean that there were more bacterial cells. Therefore, studies evaluating only bacterial richness (number of different species in a community) would not provide reliable information as to antibacterial effectiveness of treatment procedures. Moreover, despite the deep coverage of HTS methods, they still do not reveal all the species occurring in the consortium. By eliminating the most dominant OTUs in the initial samples during treatment, the small amount of DNA in the post-treatment samples can favour the detection of different OTUs (apparently increasing the richness), which may not have been detected in S1 because they were less dominant or rare. Even traces of DNA contaminants in the reagents may be amplified in low-DNA samples [34, 35]. This may help explain the appearance of some taxa in post-preparation samples, including *Acinetobacter*, *Agrobacterium*, *Pseudomonas*, and *Staphylococcus*, which are common contaminants observed in water and reagents used in molecular analyses [34, 36–38]. Abundance (and dominance) takes into account the proportion of the number of reads in a given sample (proportion of each species within a community), which may have high or low total bacterial counts. Therefore, the most plausible explanation for increased richness in many post-preparation samples is elimination of the most dominant OTUs by treatment in the initial samples and the competition of DNA targets for the PCR primers in post-preparation samples that have a low DNA content. Another reason may have been the limitations of the paper point approach (as discussed below) in providing standardized samples from the canals in two different time points. Although one cannot discard the possibility of contamination, this is highly unlikely to have occurred because of the careful aseptic conditions and the fact that the canals were usually filled with antimicrobial solutions in the time–space between S1, S2, and S3 taking, with no favourable conditions for the arrival of other species.

HTS technologies have the main advantage of promoting deep sample coverage, which allows for detection of bacteria that occur in low abundance [14]. In this study, a large number of sequences were evaluated per individual case, and the rarefaction curves obtained indicated that a good depth of coverage was reached. However, the differences between observed and estimated OTUs suggest that some taxa remained undetected and may have accounted for the discrepancies in richness findings.

This study has limitations other than the richness of artifacts discussed above. One of them includes conventional paper point sampling, which can pick bacterial cells floating in the main canal lumen, attached to the canal walls, and possibly in the immediate vicinity of the main canal lumen [14]. However, areas more distant from the main canal can harbour residual bacteria that escaped the effects of chemomechanical procedures [39, 40], which may not be recovered by paper points. These areas may require special approaches to be properly sampled, including the cryogenic grinding method [41]. Nevertheless, an important impediment to using cryopulverization is that it is a destructive approach that does not permit samples to be taken at different time points. Another possible limitation is the fact that most DNA-based detection methods do not distinguish vital from dead cells. This is of particular concern when evaluating samples taken immediately after preparation, as DNA from cells that recently died might still be detected [42]. However, it is highly likely that NaOCl used during root canal irrigation may rapidly degrade (rendering undetectable) or wash away free DNA released by dead bacteria [43]. Even so, for a most comprehensive analysis of the endodontic infection after treatment, a combination of molecular methods with advanced culturing techniques might be recommended [30].

## Conclusion

Both treatment protocols caused drastic changes in the root canal diversity, with no apparent significant differences between them. Most of the dominant taxa involved in the primary infection and probably in the aetiology of apical periodontitis were eliminated or substantially reduced. Other taxa found as dominants in post-preparation samples were not dominant or even found in the initial samples. Because post-preparation samples usually have no or very low amounts of bacteria, these taxa exclusively revealed in these samples are conceivably in low numbers and, as such, they may have minor if any relevance. Further studies focusing on these issues are necessary for clarification. Of the several most dominant taxa in the initial infection, only members of *Fusobacterium*, *Porphyromonas*, *Staphylococcus*, and *Bacteroidaceae* [G-1] were found as dominants in post-preparation samples.

## Data Availability

The supporting data for the study can be accessed at Mendeley Data, V2, doi: 10.17632/8f5nmkxxrt.2
